# Dual-functional micro- to nano-CFR-PEEK: promoting Hippo-mediated osseointegration while inhibiting *P. gingivalis* and osteoclastogenesis

**DOI:** 10.1093/rb/rbag133

**Published:** 2026-06-13

**Authors:** Yuxuan Lin, Huilian Zhang, Shiqiang Wang, Ge Yin, Yongmin Wu, Xinze Weng, Lixia Lin, Yan Chen, Yingzhen Lai, Jingjing Su

**Affiliations:** Stomatological Hospital of Xiamen Medical College; School of Stomatology of Xiamen Medical College; Xiamen Key Laboratory of Stomatological Disease Diagnosis and Treatment; Engineering Research Center of Stomatological Biomaterials, Fujian Province University, Xiamen Medical College, Xiamen 361000, China; Stomatological Hospital of Xiamen Medical College; School of Stomatology of Xiamen Medical College; Xiamen Key Laboratory of Stomatological Disease Diagnosis and Treatment; Engineering Research Center of Stomatological Biomaterials, Fujian Province University, Xiamen Medical College, Xiamen 361000, China; Stomatological Hospital of Xiamen Medical College; School of Stomatology of Xiamen Medical College; Xiamen Key Laboratory of Stomatological Disease Diagnosis and Treatment; Engineering Research Center of Stomatological Biomaterials, Fujian Province University, Xiamen Medical College, Xiamen 361000, China; Stomatological Hospital of Xiamen Medical College; School of Stomatology of Xiamen Medical College; Xiamen Key Laboratory of Stomatological Disease Diagnosis and Treatment; Engineering Research Center of Stomatological Biomaterials, Fujian Province University, Xiamen Medical College, Xiamen 361000, China; Stomatological Hospital of Xiamen Medical College; School of Stomatology of Xiamen Medical College; Xiamen Key Laboratory of Stomatological Disease Diagnosis and Treatment; Engineering Research Center of Stomatological Biomaterials, Fujian Province University, Xiamen Medical College, Xiamen 361000, China; Stomatological Hospital of Xiamen Medical College; School of Stomatology of Xiamen Medical College; Xiamen Key Laboratory of Stomatological Disease Diagnosis and Treatment; Engineering Research Center of Stomatological Biomaterials, Fujian Province University, Xiamen Medical College, Xiamen 361000, China; Stomatological Hospital of Xiamen Medical College; School of Stomatology of Xiamen Medical College; Xiamen Key Laboratory of Stomatological Disease Diagnosis and Treatment; Engineering Research Center of Stomatological Biomaterials, Fujian Province University, Xiamen Medical College, Xiamen 361000, China; Stomatological Hospital of Xiamen Medical College; School of Stomatology of Xiamen Medical College; Xiamen Key Laboratory of Stomatological Disease Diagnosis and Treatment; Engineering Research Center of Stomatological Biomaterials, Fujian Province University, Xiamen Medical College, Xiamen 361000, China; Stomatological Hospital of Xiamen Medical College; School of Stomatology of Xiamen Medical College; Xiamen Key Laboratory of Stomatological Disease Diagnosis and Treatment; Engineering Research Center of Stomatological Biomaterials, Fujian Province University, Xiamen Medical College, Xiamen 361000, China; Stomatological Hospital of Xiamen Medical College; School of Stomatology of Xiamen Medical College; Xiamen Key Laboratory of Stomatological Disease Diagnosis and Treatment; Engineering Research Center of Stomatological Biomaterials, Fujian Province University, Xiamen Medical College, Xiamen 361000, China

**Keywords:** carbon fiber-reinforced polyetheretherketone, sulfonation-nitration, micro-to-nano hierarchical topography, osseointegration, angiogenesis, osteoclast inhibition, antibacterial, Hippo-YAP/TAZ

## Abstract

To construct a biomimetic hierarchical topography on carbon fiber-reinforced polyetheretherketone (CFR-PEEK) to enhance multifaceted biological performance, CFR-PEEK substrates were modified via a one-step nitration-sulfonation treatment using mixed nitric and sulfuric acids at various volume ratios. The resulting surface topographies were systematically evaluated for *in vitro* bioactivity (osteogenesis, angiogenesis, osteoclast inhibition and antibacterial activity) and molecular mechanisms via RNA sequencing, alongside *in vivo* validation using a rat cranial defect model. A nitric-to-sulfuric acid ratio of 1:4 yielded a unique nanocluster-interspersed three-dimensional porous architecture. Upon cell-material interaction, this tailored surface upregulated osteogenic gene expression (*RUNX-2*, *ALP* and *OCN*) by suppressing the Hippo pathway and driving YAP/TAZ nuclear translocation. The 1:4 topography robustly enhanced endothelial cell migration and tube formation, while disrupting actin rings to suppress osteoclastogenesis (reducing *TRAP* and *Cathepsin K* expression) and selectively inhibiting *Porphyromonas gingivalis* colonization. *In vivo* evaluations confirmed accelerated bone mineralization, minimized osteoclast activation and enhanced functional vascularization at the bone-implant interface. Overall, the optimized 1:4 mixed-acid etching endows CFR-PEEK with a bioactive micro- and nano-hierarchical surface. By precisely orchestrating the Hippo-YAP/TAZ mechanotransduction axis, this unique architecture balances osteogenesis, angiogenesis, anti-osteoclastogenesis and targeted antibacterial activity, offering a highly efficient and clinically translatable surface modification strategy for next-generation dental implants.

## Graphical abstract

**Graphical Abstract:**
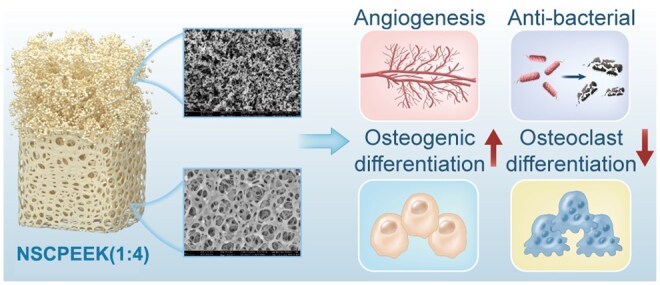

## Introduction

Dental implants require rapid, stable and long-term osseointegration for clinical success. An ideal implant material must possess both excellent biocompatibility and robust mechanical properties. Traditional metallic implants, such as titanium (Ti) and its alloys, offer high mechanical strength. However, their elastic moduli (∼110 GPa) far exceed that of bone (∼20 GPa), which can trigger stress shielding and subsequent implant loosening [1, 2]. While zirconia offers superior esthetics, its intrinsic brittleness and high modulus (∼215 GPa) pose a risk of catastrophic fracture under dynamic masticatory loads [3, 4]. Polyetheretherketone (PEEK) exhibits an elastic modulus (3–4 GPa) comparable to that of trabecular bone and superior compatibility with magnetic resonance imaging (MRI) [5]. However, its mechanical strength remains lower than that of cortical bone and dentin, making it challenging to withstand functional masticatory loads. Carbon fiber-reinforced polyetheretherketone (CFR-PEEK), with 30 wt% carbon fibers, significantly enhances mechanical strength while maintaining a bone-like elastic modulus and good biocompatibility [6]. CFR-PEEK outperforms other modified PEEK formulations (e.g., GFR-PEEK, HA-PEEK) in mechanical properties, with a tensile strength of 256.1 MPa and robust flexural performance [7, 8]. Its modulus can be tuned by adjusting the carbon fiber fraction to better match dental implant biomechanics [9]. However, its biological inertness, hydrophobicity and lack of antimicrobial activity limit cell adhesion and osseointegration, predisposing it to implant failure [10]. Thus, surface modification is essential to achieve successful biological integration and outperform alternative materials [11, 12].

Natural bone is a hierarchically organized composite, featuring dense cortical layers and porous cancellous structures that synergistically provide structural support and a vascularized niche [13–15]. Mimicking this micro- and nano-hierarchical architecture promotes cell differentiation and nutrient delivery, thereby accelerating osseointegration, while simultaneously inhibiting bacterial biofilm formation [14, 16]. Recent breakthroughs in bioactive implant materials have demonstrated that rationally designed hierarchical micro-/nano-topographical structures can effectively orchestrate osteoimmune regulation, macrophage polarization, angiogenesis and osteogenic differentiation, thereby creating a favorable regenerative microenvironment and significantly enhancing bone–implant osseointegration and tissue regeneration [17–21].

Previous studies have utilized sulfonation to introduce porous structures and sulfonic acid groups (-SO_3_H) on PEEK surfaces to enhance hydrophilicity [22, 23]. However, conventional concentrated sulfuric acid etching is often excessively corrosive, compromising the material’s structural framework and mechanical integrity [24]. Conversely, nitric acid treatment is milder and can introduce nitro (-NO_2_) groups, which facilitate biomolecule adsorption and promote osteogenesis [24, 25]. Compared with other reported CFR-PEEK surface modification methods (plasma treatment, laser etching and coating modification) [26], nitration-sulfonation chemical modification has unique advantages: plasma treatment improves hydrophilicity temporarily without stable hierarchical structures; laser etching forms micro- and nano-topographies but causes thermal damage; coating modification enhances osteogenesis but has poor coating-substrate adhesion and easy peeling under masticatory load [27–29]. Leveraging the complementary properties of nitration and sulfonation, we adjusted the mixed ratio of nitric and sulfuric acids to achieve synergistic performance optimization. This approach enables precise control over micro- and nano-hierarchical structures without compromising the material’s bulk mechanical properties. Furthermore, it simultaneously significantly increases the specific surface area and introduces dual-functional groups (-SO_3_H and -NO_2_), which synergistically enhance protein and ion adsorption, thereby accelerating fibrin clot formation. This cascade of events subsequently promotes osteogenic differentiation of bone marrow mesenchymal stem cells through cellular mechanotransduction, significantly enhancing the material’s osseointegration capacity [30, 31].

Successful osseointegration is not merely bone formation; it is a complex biological process centered on maintaining a dynamic equilibrium between osteogenesis and osteoclastogenesis [14, 32], while ensuring adequate angiogenesis and effective immunomodulation to prevent infection. The implant-induced local microtrauma activates RANKL signaling, prompting osteoclasts to clear the damaged bone matrix and create a ‘biological space’ for subsequent repair. Subsequent angiogenesis delivers oxygen and nutrients to the repair site, laying the foundation for osteoblast migration and proliferation [32]. Following neovascularization, osteoblasts are recruited to the defect area, where key signaling pathways such as BMP-2/Smad and Wnt/β-catenin drive collagen deposition and matrix mineralization [33]. Disruption at any stage, whether due to excessive osteoclastic activity, inadequate vascular networks or inflammation triggered by bacterial infection that upregulates the RANKL/OPG ratio, can destabilize bone repair equilibrium, leading to dysregulated resorption, central necrosis or even implant loosening [34]. To synergistically regulate this complex process, researchers have proposed constructing biomimetic hierarchical micro‑to‑nano structures that mimic natural bone tissue [6]. Such materials facilitate multilevel signaling coordination through two primary mechanisms: mechanical coupling (where different-scale topologies regulate pathways like integrin-FAK-ERK and YAP/TAZ) [35, 36] and chemical coupling (where surface chemical groups or nanoclusters directly exert biological functions) [37]. This approach simultaneously induces osteogenic differentiation, suppresses osteoclastogenesis, promotes vascularization and inhibits bacterial proliferation, thereby providing an ideal material platform for achieving ‘closed loop’ bone regeneration. Yang *et al.* [38] discovered that micro-/nano-meshed titanium metal activates the Src-ROCK signaling pathway, promoting anti-inflammatory M2 macrophage polarization, which in turn achieves synergistic regulation of key osteogenic genes (*RUNX-2*, *ALP*) and angiogenic genes (*VEGF*, *CD31*). Yu *et al.* [39] introduced amino groups onto CFR-PEEK surfaces, activating the focal adhesion kinase (FAK) signaling pathway through amino functionalization and upregulating the expression of integrin α2 in osteoblasts.

**Figure 1:**
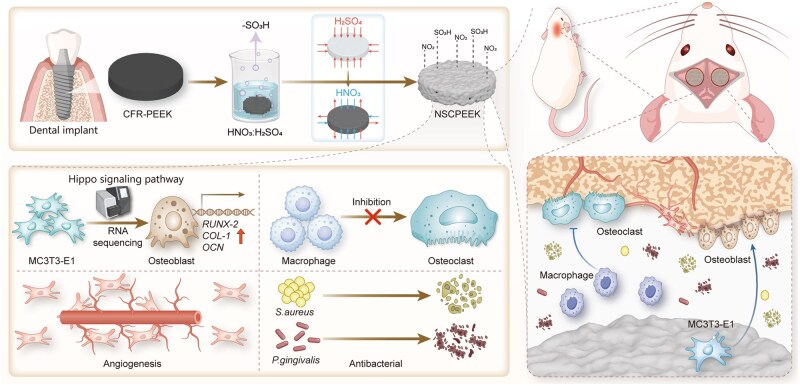
Schematic diagram of the main experimental process.

Based on these principles, this study employed CFR-PEEK as a substrate, modifying its surface through a synergistic nitration-sulfonation treatment to construct a multi-level hierarchical micro‑to‑nano structure. This strategy was designed to simultaneously achieve high mechanical strength, excellent osteogenic/angiogenic induction capabilities, antibacterial properties and inhibition of osteoclast activity. To validate this approach, systematic *in vitro* experiments were conducted to evaluate cellular responses and antimicrobial performance. Concurrently, high-throughput sequencing was employed to decipher the molecular mechanisms regulating osteogenic differentiation. Finally, *in vivo* animal experiments were conducted to validate the overall efficacy of this approach in promoting osseointegration (Figure 1).

## Materials and methods

### Material preparation and characterization

#### Materials

The following analytical-grade chemical reagents were used: acetone (≥99.5% purity) and anhydrous ethanol (≥99.7% purity) and 75% ethanol (Sinopharm Chemical Reagent Co., Ltd., China); nitric acid (65 wt%, Sinopharm Chemical Reagent Co., Ltd., China); and concentrated sulfuric acid (95–98 wt%, Xilong Chemical, China). The substrate material was 30% carbon fiber-reinforced polyetheretherketone (CFR-PEEK, PEEK matrix molecular weight ≈ 80 000 g/mol, Roechling, Germany). Four distinct samples were prepared based on the assay requirements: (1) Ø 13 mm × 2 mm (physicochemical characterization and *in vitro* cell/bacterial experiments); (2) Ø 30 mm × 2 mm (real-time quantitative PCR, western blot); (3) Ø 10 mm × 8 mm (mechanical testing); (4) Ø 5 mm × 1 mm (*in vivo* animal experiments). Prior to use, all samples underwent sequential ultrasonic cleaning in acetone, anhydrous ethanol and deionized water for 10 min each, followed by air-drying.

#### Mixed-acid modification of PEEK surfaces

CFR-PEEK specimens were treated with mixed HNO_3_ (65%) and H_2_SO_4_ (98%) at volume ratios of 0:1, 1:0, 1:1, 1:2, 1:4, 2:1 and 4:1. Samples were immersed in 30 mL of the respective mixed acid at room temperature for 6 min under magnetic stirring, followed by a 5-min rinse with deionized water. Subsequently, hydrothermal treatment was conducted at 120°C for 4 h. Finally, the samples were thoroughly rinsed three times with distilled water (10 min for the first two washes and 1 h for the final wash) until the wash water reached a neutral pH (7.0). Untreated PEEK samples served as the control group (C). Prior to biological assays, all samples were sterilized by immersion in 75% ethanol for 30 min, followed by UV irradiation for 1 h on each side.

#### Physicochemical property testing

The surface morphology was characterized by using field-emission scanning electron microscopy (FE-SEM, Magellan400). Surface functional groups were identified by attenuated total reflectance Fourier-transform infrared spectroscopy (ATR-FTIR, Thermo Fisher), while elemental composition and chemical states were analyzed using X-ray photoelectron spectroscopy (XPS, Thermo Fisher K-Alpha). Mechanical properties, specifically elastic modulus and compressive strength, were evaluated using a universal testing machine (Shimadzu AGS-X). Surface roughness was quantified by atomic force microscopy (AFM, Bruker Dimension Icon). Static water contact angles were determined using a contact angle meter (Data Physics OCA20) to assess surface wettability. The pH variations of the immersion solutions were monitored at 1, 3, 7, 14 and 28 days using a pH meter (Seven Compact S210 B, Mettler Toledo).

### *In vitro* cell experiments

#### Cell culture and induction

MC3T3-E1 (GNM15), RAW264.7 (TCM13) and HUVEC (EA.hy926) cell lines were used. MC3T3-E1 and RAW264.7 cells were cultured in α-MEM and high-glucose DMEM, respectively, while HUVECs were maintained in low-glucose DMEM. All media were supplemented with 10% FBS and 1% penicillin-streptomycin. Cells were incubated at 37°C with 5% CO_2_, passaged upon reaching 85% confluence and used between the third and fifth passages. The osteogenic induction medium contained 0.1 µM dexamethasone, 10 mM sodium β-glycerophosphate and 50 µg/mL ascorbic acid. The osteoclast induction medium was supplemented with 50 ng/mL RANKL.

#### Cell proliferation and adhesion

Cells were seeded onto the modified surfaces at specific densities (MC3T3-E1: 5 × 10^4^ cells/well; RAW264.7 and HUVEC: 3 × 10^4^ cells/well). Proliferation was evaluated at Days 1, 3 and 5 using the CCK-8 assay by measuring the absorbance at 450 nm. For adhesion morphology, MC3T3-E1 (cultured for 24 h) and RAW264.7 cells (cultured for 5 days) were fixed and stained with DAPI (for nuclei) and rhodamine-phalloidin (for the cytoskeletal F-actin). Cell morphology was observed via confocal laser scanning microscopy (CLSM).

#### ALP staining and activity assay

MC3T3-E1 cells (2 × 10^4^ cells/well) were cultured in osteogenic medium for 7 and 14 days, followed by staining with a BCIP/NBT kit (C3206, Beyotime, China) and macroscopic imaging. For quantitative analysis of early osteogenic differentiation, cells (6 × 10^4^ cells/well) were cultured, and intracellular ALP activity was quantified and normalized to total protein content determined by a BCA Protein Assay Kit (B5001, Lanbolide, China).

#### Alizarin red staining and semi-quantitative analysis

MC3T3-E1 cells (2 × 10^4^ cells/well) were osteogenically induced for 14 and 21 days. Extracellular matrix mineralization was evaluated using Alizarin Red S (A5533, Sigma, USA) staining. For semi-quantification, the stained calcium nodules were destained using 10% cetylpyridinium chloride (C9890, Solarbio, China) and the absorbance of the extract was measured at 560 nm.

#### TRAP quantification

RAW264.7 cells (1 × 10^5^ cells/well) were cultured in osteoclast induction medium for 5 days. TRAP activity was quantified using a tartrate-resistant acid phosphatase assay kit (P0332, Beyotime, China) and normalized to total protein concentration (BCA assay, P0010, Beyotime, China).

#### Scratch assay

HUVECs (1 × 10^5^ cells/well) were seeded and incubated until 90% confluence. A linear scratch was created using a sterile 200 μL pipette tip. Cellular migration was documented microscopically at 0, 12 and 24 h, and the scratch closure area was quantified using ImageJ software.

#### Tube formation assay

Pre-chilled 96-well plates were coated with 50 μL/well of Matrigel and incubated at 37°C for 30 min to polymerize. HUVECs (3 × 10^4^ cells/well), previously co-cultured with the material samples for 3 days, were seeded onto the Matrigel. After 6 h of incubation, the formation of capillary-like networks was photographed and analyzed using ImageJ software.

#### RT-qPCR

MC3T3-E1 (6 × 10^4^ cells/well, 7 and 14 days osteogenic induction), RAW264.7 (1 × 10^5^ cells/well, 5 days osteoclast induction) and HUVECs (1 × 10^5^ cells/well, 3 and 7 days culture) were utilized. Total RNA was extracted using TRIzol reagent. Using GAPDH as the housekeeping gene, specific primers (Table 1) were employed to quantify the relative mRNA expression of Hippo pathway-related genes (*LATS1*, *SAV1*), extracellular matrix and osteogenic genes (*CYR61*/*CCN1*, *CTGF*/*CCN2*, *FN1*, *Serpine1*), osteogenic (*RUNX-2, ALP, COL-1, OCN, OPN*), osteoclastic (*MMP-9, Cathepsin K, TRAP, NFATc1*) and angiogenic genes (*VEGF, ANG-1, FGFR-1, HIF-1α*).

**Table 1:** Primers for real-time fluorescent quantitative PCR in this study.

Gene	Primer
*GAPDH* (Mus musculus)	AAATGGTGAAGGTCGGTGTGAAC CAACAATCTCCACTTTGCCACTG
*RUNX-2*	AACGATCTGAGATTTGTGGGC CCTGCGTGGGATTTCTTGGTT
*ALP*	CCAACTCTTTTGTGCCAGAGA GGCTACATTGGTGTTGAGCTTTT
*COL-1*	GCTCCTCTTAGGGGCCACT CCACGTCTCACCATTGGGG
*OCN*	CTGACCTCACAGATCCCAAGC TGGTCTGATAGCTCGTCACAAG
*OPN*	AGCAAGAAACTCTTCCAAGCAA GTGAGATTCGTCAGATTCATCCG
*LATS1*	CCGGCGTGTGTCCCC GGAGCCCGTCCCCCA
*SAV1*	CCTGTGCTCCGAGATATGACC CAGCATTCCCTGGTACGTGT
*CYR61* (*CCN1*)	CACTGAAGAGGCTTCCTGTCTT GGGTTGAAAAGAACTCGCGG
*CTGF* (*CCN2*)	GAGAACTGTGTACGGAGCGT GGTGCACCATCTTTGGCAGTG
*FN1*	TTCCATCTCCTTACCGGCGT TGAGCATCTTGAGTGGATGGG
*Serpine1*	GATGACCACAGCGGGGAAAA GAGCTGTGCCCTTCTCATTG
*MMP9*	ATGTCACTTTCCCTTCACCTTC TGCCGTCCTTATCGTAGTCA
*Cathepsin K* (*CTSK*)	AATACCTCCCTCTCGATCCTACA TGGTTCTTGACTGGAGTAACGTA
*TRAP*	CACTCCCACCCTGAGATTTGT CATCGTCTGCACGGTTCTG
*NFATc1*	CCGTCACATTCTGGTCCATAC
	TTCATTCTCCAAGTAACCGTGTAG
*GAPDH* (Human)	ACCCACTCCTCCACCTTTGAC TCCACCACCCTGTTGCTGTAG
*VEGF*	CGCTTACTCTCACCTGCTTCTG TCCAACAATGTGTCTCTTCTCTTCG
*ANG-1*	ACCCTCACAGAGAAAACCTAAG GACGACGGAAAATTGACTGATC
*FGFR-1*	CTGGGAGAGGGCTGCTTTGG CACTTTGGTCACACGGTTGGG
*HIF-1α*	AGGACACAGATTTAGACTTGGAGATG CAGTGGTAGTGGTGGCATTAGC

#### RNA-seq sequencing

MC3T3-E1 cells (6 × 10^4^ cells/well) were cultured on different surfaces in osteogenic medium for 14 days. Total RNA was extracted, and paired-end RNA sequencing was performed on an Illumina sequencing platform (NovaSeq 6000) by Majorbio Bio-Pharm Technology Co., Ltd. Differentially expressed genes (DEGs) were identified, and hypergeometric tests were applied for Gene Ontology (GO) and Kyoto Encyclopedia of Genes and Genomes (KEGG) pathway enrichment analyses.

#### Western blot

Total protein was extracted from MC3T3-E1 cells after 14 days of osteogenic induction, and concentrations were determined via BCA assay. Equal amounts of protein (5 μg/lane) were separated by 8% SDS-PAGE and transferred onto PVDF membranes (IPVH00010, Sigma, USA). Membranes were blocked with 5% skim milk for 1 h, incubated with primary antibodies overnight at 4°C, and subsequently probed with secondary antibodies for 1 h at room temperature. Protein bands were visualized using an enhanced chemiluminescence (ECL) substrate and semi-quantified with ImageJ software.

#### Immunofluorescence imaging of YAP

MC3T3-E1 cells cultured on C and 1:4 samples for 24 h were fixed with 4% paraformaldehyde (15 min), permeabilized with 0.3% Triton X-100 (10 min), and blocked with 5% BSA (1 h). Cells were incubated with an anti-YAP primary antibody (1:300, #14074 T, Cell Signaling Technology, USA) overnight at 4°C followed by an Alexa Fluor 594-conjugated secondary antibody (1:500, #550043, Zenbio, China) for 1 h. Nuclei were counterstained with DAPI. YAP subcellular localization was observed via CLSM.

### *In vitro* bacterial experiments

#### Bacterial culture

*Staphylococcus aureus* (ATCC 25923) was cultured aerobically in Lysogeny Broth (LB) medium at 37°C. *Porphyromonas gingivalis* (ATCC 33277) was cultured anaerobically in Brain Heart Infusion (BHI) broth supplemented with 5 mg/L hemin, 1 mg/L vitamin K3 and 5% yeast extract at 37°C. Both bacterial suspensions were adjusted to an optical density (OD600) equivalent to 1 × 10^8^ CFU/mL for subsequent experiments.

#### Bacterial viability staining

Samples were co-cultured with 1 mL of bacterial suspension (1 × 10^8^ CFU/mL) for 24 h. Biofilms were stained using the LIVE/DEAD BacLight Bacterial Viability Kit for 30 min. Under CLSM, SYTO-9 stained live bacteria green, while propidium iodide (PI) stained cells with compromised membranes (dead bacteria) red.

#### Assessment of bacterial survival rates

Materials were inoculated with 1 mL of bacterial suspension at 37°C. The survival rates of *S. aureus* (after 24 h) and *P. gingivalis* (after 72 h) were evaluated using the standard agar plate counting method. The survival rate was calculated as (T/C) × 100%, where C and T denote the average colony counts on untreated CFR-PEEK and acid-treated samples, respectively.

#### Biofilm CCK-8 assay

Materials were cultured in a 1 × 10^8^ CFU/mL bacterial suspension for 24 h to form biofilms. CCK-8 reagent was added, and absorbance was measured at 450 nm to assess metabolic activity.

### *In vivo* osteogenesis experiment

This animal study was approved by the Animal Ethics Committee of Xiamen Medical College (Approval No.: 20250528003) and strictly adhered to the relevant guidelines of the Chinese Association for Laboratory Animal Science. A Ø 5 mm rat cranial defect model was used to evaluate the osseointegration of the material. Twelve healthy male SD rats (4 weeks old, weighing 200–240 g) were randomly divided into four groups. Bilateral cranial defects were created and implanted with the respective materials. At 8 weeks post-implantation, the rats were euthanized and the craniums were harvested and fixed in 4% paraformaldehyde. Samples underwent micro-CT scanning to quantify bone mineral density (BMD) and bone volume fraction (BV/TV). For histological evaluation, nondecalcified samples were resin-embedded and subjected to Van Gieson (VG) staining. Meanwhile, EDTA-decalcified samples were sectioned for Hematoxylin and Eosin (H&E), Masson’s trichrome and TRAP staining. Furthermore, immunohistochemistry for OCN and CD31 was performed to evaluate osteogenesis and angiogenesis, respectively.

### Statistical analysis

All quantitative data are expressed as mean ± standard deviation (SD). *In vitro* assays were performed in at least triplicate (*n* ≥ 3 independent replicates per group). For *in vivo* experiments, three animals per group were utilized, with two implantation sites per animal (*n* = 6 implants per group). GraphPad Prism 8 was used for statistical analysis. After confirming data normality and homogeneity of variance, comparisons between groups were analyzed using one-way or two-way ANOVA followed by appropriate *post hoc* tests. Statistical significance was defined as *P *< 0.05.

## Results

### Surface morphology and physicochemical properties

**Figure 2:**
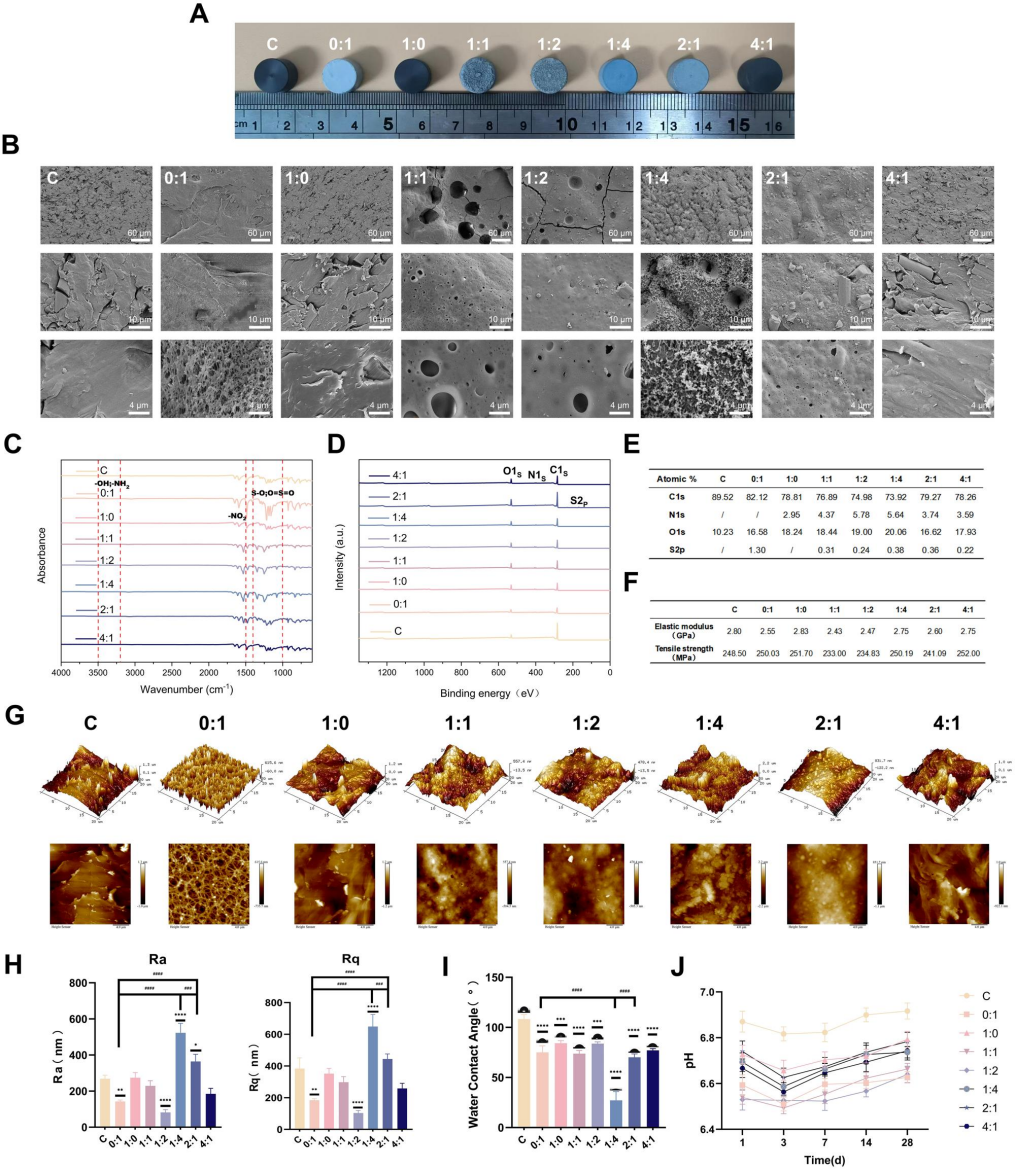
Surface characteristics of the modified CFR-PEEK substrates. (**A**) Gross photographs of the eight experimental samples (C, 0:1, 1:0, 1:1, 1:2, 1:4, 2:1 and 4:1). (**B**) SEM images of each sample at varying magnifications (300×, scale bar = 60 μm; 2000×, 10 μm; 10 000×, 4 μm). (**C**) FTIR spectra and (**D**) XPS full spectra of the samples. (**E**) Elemental composition ratios derived from XPS analysis. (**F**) Elastic modulus and compressive strength. (**G**) AFM 3D topographical images and (**H**) corresponding surface roughness parameters (*Ra* and *Rq*). (**I**) Water contact angles. (**J**) The pH variations of the immersion solutions for each sample after 1, 3, 7, 14 and 28 days. Data are presented as mean ± SD (*n* = 3). *P < 0.05, **P < 0.01, ***P < 0.001, ****P < 0.0001 vs. the C (CFR-PEEK) control group; #*P *< 0.05, ##*P *< 0.01, ###*P *< 0.001, ####*P *< 0.0001 among the 0:1, 1:4 and 2:1 groups.

The surface color and microstructure of CFR-PEEK were systematically modulated by altering the HNO_3_/H_2_SO_4_ volume ratio, as evidenced in Figure 2A and B. The untreated sample exhibited a black coloration. Treatment with pure sulfuric acid (0:1) yielded a white surface with uniform three-dimensional pores, while treatment with pure nitric acid (1:0) yielded a black, corroded landscape with successful nitration (Figure 2C). As the sulfuric acid ratio increased (1:1 → 1:4), the color gradually lightened and the surface morphology transitioned from irregular nanopores (1:1, 1:2) to a three-dimensional porous structure composed of nanocluster aggregates (1:4). Conversely, increasing the nitric acid ratio (1:1 → 4:1) darkened the color. The 2:1 group exhibited numerous fine nanopores, while the 4:1 group reverted to a distinct corrosion landscape. Based on these observations, four typical surface morphologies can be summarized: corrosion landforms (1:0, 4:1), nanopore structures (1:1, 1:2, 2:1), three-dimensional porous structures (0:1) and nanocluster-composite three-dimensional porous structures (1:4). FTIR analysis (Figure 2C) revealed that samples with ≥50% sulfuric acid content (0:1, 1:1, 1:2, 1:4) exhibited characteristic sulfonic acid group (S-O, O=S=O) peaks in the 1000–1400 cm^−1^ region, whereas samples with high nitric acid content (1:0, 2:1, 4:1) showed only weak nitro absorption in the 1400–1500 cm^−1^ region. All mixed-acid groups exhibited coexisting sulfonic and nitro groups, with no abnormal vibrations detected for hydroxyl or amino groups, confirming the successful incorporation of these functional groups into the polymer backbone. XPS analysis (Figure 2D and E) revealed characteristic peaks at C1s (286.00 eV) and O1s (532.87 eV) for all samples. Surfaces treated with sulfonation (0:1) and mixed acids exhibited an S2p signal (168.25 eV), while nitration (1:0) and mixed-acid groups showed an N1s peak (406.23 eV). The mixed-acid process resulted in co-doping of N and S, characterized by significantly lower S content (0.22–0.38%) than that of the monosulfuric acid group (1.30%), while the N content (3.59–5.78%) was higher than that in the mononitric acid group (2.95%), indicating a synergistic modification effect.

As depicted in Figure 2F, mechanical property tests indicated limited effects of different acid treatments on elastic modulus and compressive strength, with overall controllable variations (elastic modulus: 2.43–2.83 GPa; compressive strength: 233.00–252.00 MPa). The 1:1 and 1:2 groups exhibited marginally lower elastic moduli (approximately 2.43–2.47 GPa), while the 1:0 group yielded the highest value (approximately 2.83 GPa). Compressive strength exceeded 250 MPa in the 1:0, 4:1, 0:1 and 1:4 groups. The surface topography assessed by AFM (Figure 2G and H) was largely consistent with the SEM observations. The untreated CFR-PEEK exhibited relatively high roughness, with an arithmetic mean (*Ra*) of 268.67 ± 19.76 nm and a root mean square (*Rq*) of 383.67 ± 68.19 nm. Treatment with pure sulfuric acid (0:1) significantly reduced the surface roughness, yielding values of *Ra *= 143.00 ± 7.81 nm and *Rq *= 184.00 ± 9.54 nm. Treatment with pure nitric acid (1:0) showed minor effects (*Ra*: 274.33 ± 28.75 nm; *Rq*: 351.67 ± 33.01 nm). Significant variations in roughness were observed among mixed-acid groups. The 1:2 composite yielded the smoothest surface, with the lowest recorded values (*Ra*: 82.17 ± 15.31 nm; *Rq*: 103.40 ± 16.46 nm). In contrast, the nanocluster-composite structure of the 1:4 group resulted in the highest roughness (*Ra*: 522.67 ± 53.15 nm; *Rq*: 648.67 ± 76.16 nm), which was substantially greater than that of the 2:1 group (*Ra*: 364.00 ± 40.58 nm; *Rq*: 444.00 ± 32.05 nm). The 1:1 and 4:1 groups exhibited intermediate roughness values. As shown in Figure 2I, the untreated group exhibited a hydrophobic surface, with a water contact angle of 108.30 ± 4.62°. In contrast, all acid-treated samples showed a significant increase in hydrophilicity (*P *< 0.05), with contact angles consistently falling below 90°. The 1:4 group exhibited the most pronounced hydrophilicity (27.13 ± 8.76°), followed by the 2:1 group (70.13 ± 3.41°). As presented in Figure 2J, during immersion, pH values of all groups remained within the range of 6.50–7.00. Group C maintained a significantly higher pH level, while groups 0:1, 1:1 and 1:2 showed relatively lower values. Each group displayed independent and irregular minor fluctuations without a unified tendency or continuous acidification.

Based on the above surface characteristics and mechanical property analysis, the 0:1, 1:4 and 2:1 groups demonstrated outstanding comprehensive physicochemical properties in terms of microstructure, roughness and hydrophilicity.

### Proliferation and *in vitro* osteogenic differentiation of MC3T3-E1 cells

**Figure 3:**
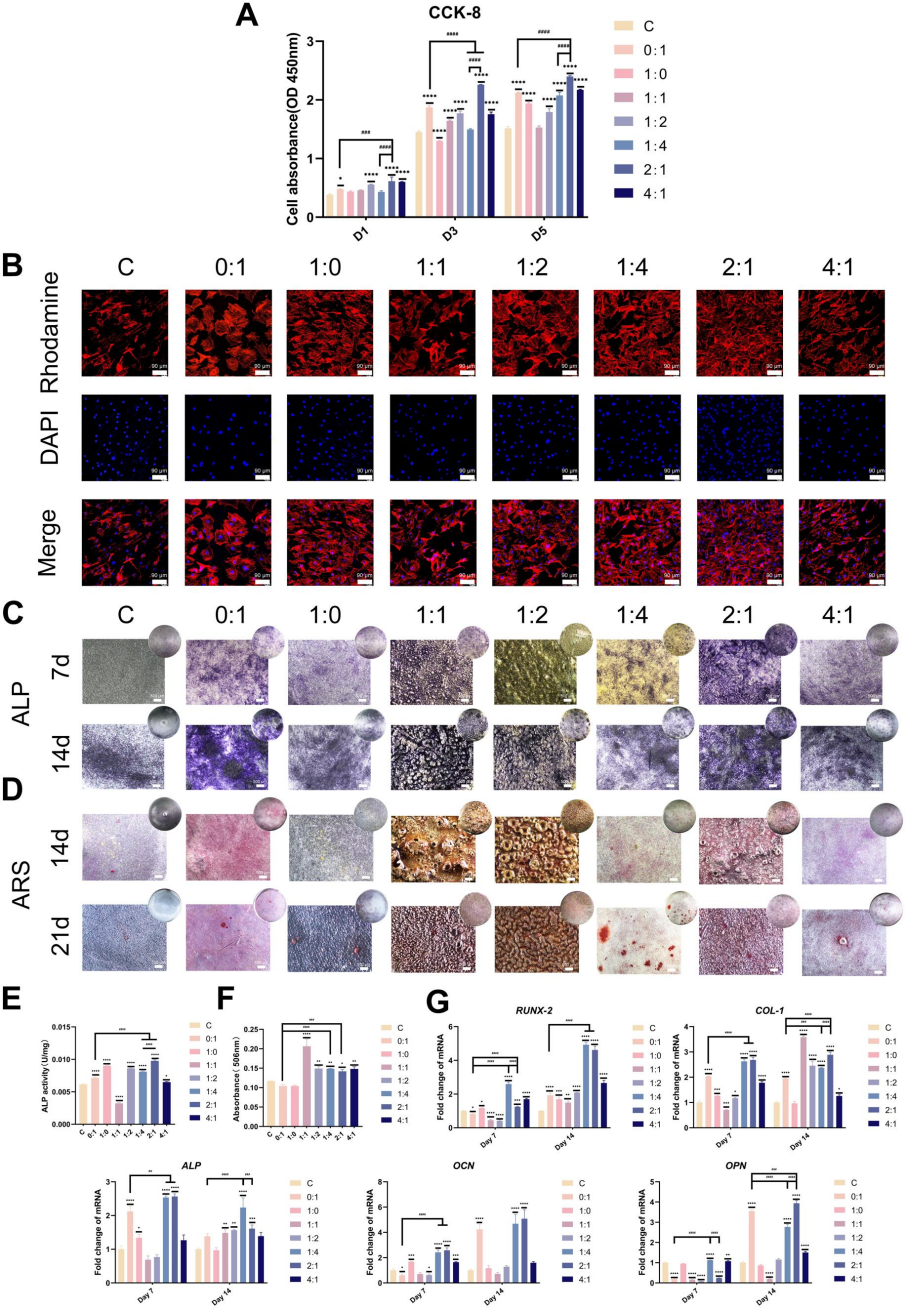
*In vitro* proliferation and osteogenic differentiation of MC3T3-E1 cells on various material surfaces. (**A**) Cytotoxicity and proliferation assay using CCK-8. (**B**) Cell morphology observed via confocal laser scanning microscopy. (**C**) ALP staining images after 7 and 14 days of culture. (**D**) Alizarin Red S (ARS) staining images after 14 and 21 days of culture. (**E**) Quantitative analysis of ALP activity and (**F)** semi-quantitative analysis of ARS staining. (**G**) Relative mRNA expression levels of osteogenesis-related genes after 7 and 14 days of culture. Data are presented as mean ± SD (*n* = 3). *P < 0.05, **P < 0.01, ***P < 0.001, ****P < 0.0001 vs. the C (CFR-PEEK) control group; #*P *< 0.05, ##*P *< 0.01, ###*P *< 0.001, ####*P *< 0.0001 among the 0:1, 1:4 and 2:1 groups.

The CCK-8 results indicated that all acid-treated material surfaces supported sustained cell proliferation, with higher absorbance values than the control group as early as Day 1 (Figure 3A), suggesting enhanced initial cell viability. By Day 5, both monosulfonated (0:1) and mononitrated (1:0) materials significantly promoted MC3T3-E1 proliferation (*P *< 0.05). In the mixed-acid groups, increasing the H_2_SO_4_ ratio (1:1 → 1:2 → 1:4) progressively enhanced cell proliferation, whereas increasing the HNO_3_ ratio (2:1 → 4:1) attenuated these effects. The overall proliferative activity exhibited the following trend: 2:1 > 0:1 > 4:1 ≈ 1:4 > 1:0 > 1:2 > 1:1 ≈ C. All groups-maintained proliferation rates above 70% of the control at Days 1, 3 and 5, demonstrating compliance with ISO 10993-5 standards and no significant cytotoxicity. Representative fluorescence confocal images of MC3T3-E1 cells cultured on material surfaces for 24 h are presented in Figure 3B, with nuclei (blue) and the cytoskeleton (red) fluorescently labeled. The control group exhibited low cell density, small cell volume and sparse pseudopodia, indicating restricted cell adhesion. Cells in acid-treated groups exhibited improved spreading with markedly elongated pseudopodia. The 0:1 group exhibited extensive cell spreading, while the 1:0, 1:4 and 2:1 groups demonstrated increased adhesion and the formation of preliminary cellular networks. Notably, cells in the 1:4 and 2:1 groups exhibited typical spindle-shaped morphology, abundant pseudopodia and the highest surface coverage, indicating favorable early cellular responses.

The early osteogenic differentiation of MC3T3-E1 cells was assessed via alkaline phosphatase (ALP) staining (Figure 3C). On Day 7, the 2:1 group exhibited the most intense staining, followed by the 0:1 group. Quantitative analysis (Figure 3E) confirmed the highest ALP activity in the 2:1 group. ALP staining significantly increased in all groups by Day 14, with the 2:1 and 0:1 groups showing comparable levels, followed by the 1:4 group, indicating sustained early osteogenic promotion. ARS staining (Figure 3D) revealed no distinct calcific nodules in any group by Day 14, though the 0:1 and 2:1 groups exhibited slightly darker staining. By Day 21, calcium nodules were observed in all samples, with the most pronounced nodule formation in the 0:1, 1:4 and 2:1 groups; notably, the 1:4 group exhibited large-particle nodules. Semi-quantitative analysis (Figure 3F) revealed a significantly higher abundance of calcium nodules in the mixed-acid-treated groups compared to the control (*P *< 0.05). RT-qPCR results (Figure 3G) showed a distinct trend in osteogenic gene expression across the test groups, with the 2:1 and 1:4 composites inducing the highest levels. Assessment of the early-stage genes (*RUNX-2, COL-1* and *ALP*) revealed that the 0:1, 1:4 and 2:1 groups exhibited the most significant upregulation, with the 1:4 group expressing approximately 2.60-fold higher levels than the control. Late-stage genes (*OCN* and *OPN*) were also significantly upregulated in the 0:1, 1:4 and 2:1 groups. The *OCN* expression in these three groups was approximately 4.67 times that of the control group, while the *OPN* expression gradient was 2:1 > 0:1 > 1:4. A comprehensive analysis revealed that the 0:1, 1:4 and 2:1 groups demonstrated optimal osteogenic effects, as indicated by cell proliferation, adhesion, ALP activity, mineralization and early/late osteogenic gene expression.

In summary, after acid etching, the 0:1, 1:4 and 2:1 groups showed the optimal effects in promoting osteogenic differentiation. RNA-seq, Western blotting (WB) and RT-qPCR were subsequently employed to elucidate the molecular mechanisms underlying osteogenic differentiation in these three groups.

### Transcriptomic study of micro‑to‑nano surface-mediated osteogenic differentiation

**Figure 4:**
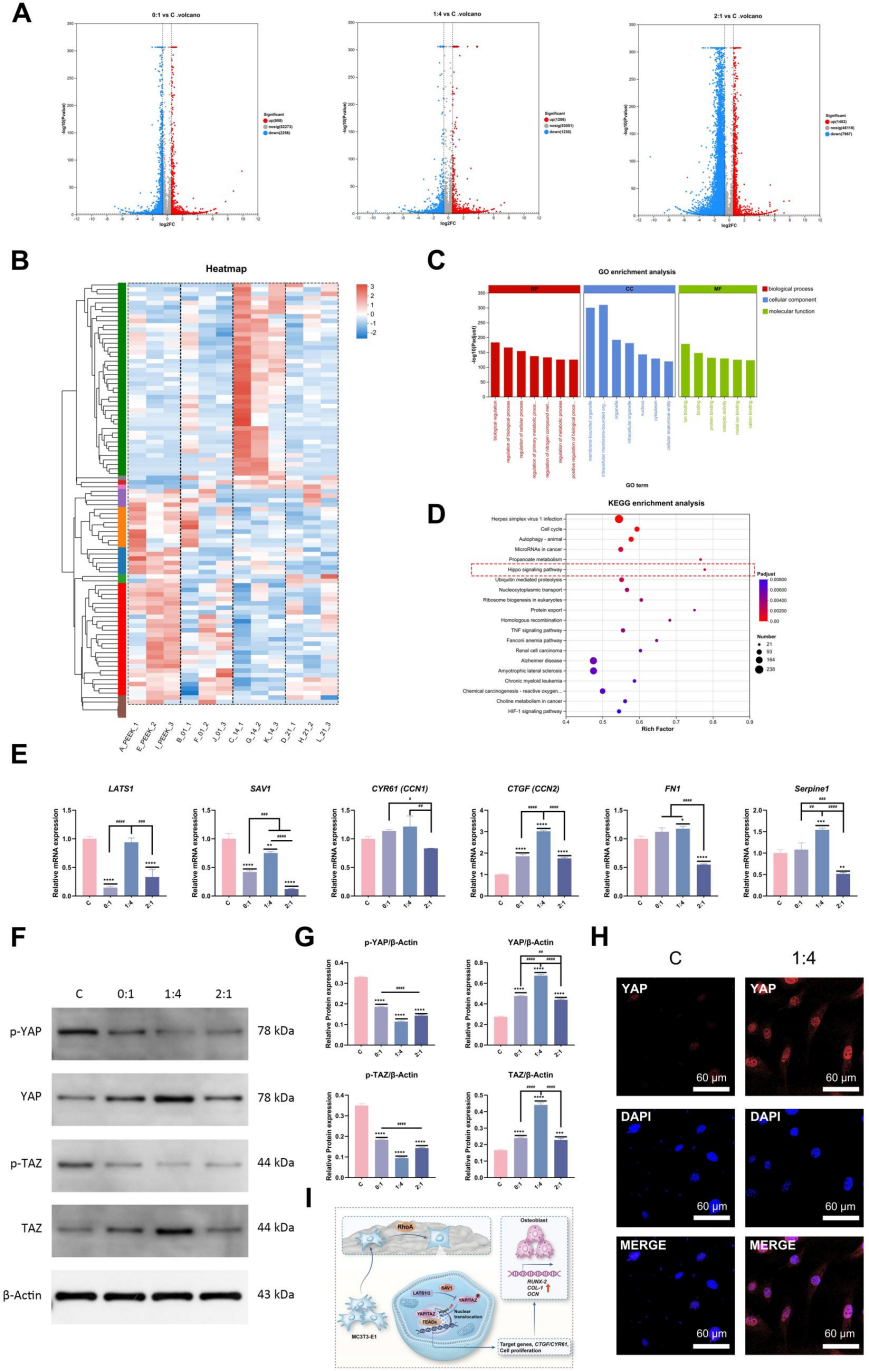
Transcriptome analysis of osteogenesis-related genes in the C, 0:1, 1:4 and 2:1 material groups. (**A**) Volcano plot showing differentially expressed genes (DEGs) in the three experimental groups compared to the control group, where three distinct data point categories represent upregulated, downregulated and nonsignificantly distinct genes, respectively. (**B**) Hierarchical clustering heatmap of DEGs across four sample groups. (**C**) Gene Ontology (GO) enrichment analysis of DEGs categorized into three primary functional domains. (**D**) KEGG pathway enrichment analysis of DEGs. (**E**) RT-qPCR validation of mRNA expression levels for Hippo-YAP/TAZ pathway-related genes. (**F**) Western blot and (**G**) corresponding semi-quantitative analyses of Hippo-YAP/TAZ pathway-related proteins. (**H**) Immunofluorescence staining of YAP in MC3T3-E1 cells on C and 1:4 groups. (**I**) Schematic diagram of the potential mechanism by which microcomposites and nanocomposites promote osteogenic differentiation. Data are presented as mean ± SD (*n* = 3). *P < 0.05, **P < 0.01, ***P < 0.001, ****P < 0.0001 vs. the C (CFR-PEEK) control group; #*P *< 0.05, ##*P *< 0.01, ###*P *< 0.001, ####*P *< 0.0001 among the 0:1, 1:4 and 2:1 groups.

RNA-seq was performed on MC3T3-E1 cells cultured on three substrates with diverse surface topographies (0:1, 1:4 and 2:1) against the control (12 samples in total, yielding 76.35 Gb of clean data, ≥5.98 Gb per sample, Q30 ≥ 96.24%). Differentially expressed genes (DEGs) were identified based on the following screening criteria: |Fold Change| ≥ 1.5 and *P *≤ 0.05. A total of 3214 (958 upregulated, 2256 downregulated), 2436 (1206 up, 1230 down) and 9369 (1402 up, 7967 down) DEGs were identified in the 0:1, 1:4 and 2:1 groups, respectively. Volcano plots (Figure 4A) and heatmap clustering (Figure 4B) revealed distinct gene expression patterns across groups, with the 1:4 group showing the most pronounced divergence from the control and exhibiting contrasting expression trends. As shown in Figure 4C, GO-enriched DEGs (*P*adj < 0.01) primarily involved biological regulation, cellular processes, membrane-bound organelles and ion/protein binding. KEGG analysis (Figure 4D) revealed the Hippo pathway as the most significantly enriched (*P*adj < 0.005). Although pathways such as HSV-1 and the cell cycle were also enriched, they showed weaker associations with osteogenesis.

Based on these findings, the Hippo pathway was identified as a key regulatory axis and subsequently validated via RT-qPCR and WB. RT-qPCR results (Figure 4E) revealed that, compared to the control group, the 0:1, 1:4 and 2:1 groups showed downregulated mRNA levels of *LATS1* and *SAV1* (core regulators of the Hippo pathway), while promoting the upregulation of downstream target genes *CYR61* and *CTGF*. Specifically, the 1:4 group exhibited partial inhibition of *LATS1* and *SAV1* expression while showing the most pronounced upregulation of *CYR61* and *CTGF*. This effect may correlate with the elevated co-expression of *FN1* and *Serpine1* in the 1:4 group, which significantly decreased in the 2:1 group. Western blot results (Figure 4F and G) further confirmed that phosphorylated YAP (p-YAP) and phosphorylated TAZ (p-TAZ) levels were significantly reduced in the experimental groups, while total YAP and TAZ protein expression increased, with the most pronounced changes observed in the 1:4 group. This indicated effective inhibition of the Hippo pathway, allowing YAP/TAZ activation.

Immunofluorescence staining (Figure 4H) was performed to detect YAP nuclear translocation in MC3T3-E1 cells. In the C group, YAP was mainly distributed in the cytoplasm, exhibiting weak nuclear fluorescence and no obvious nuclear translocation. In the 1:4 group, YAP predominantly translocated into the nucleus, as evidenced by significantly enhanced nuclear fluorescence intensity.

Building upon the above findings, we propose the following mechanism (Figure 4I): The microscale/nanoscale surface topography of the 1:4 group inhibits LATS1/2 kinase activity via the focal adhesion-RhoA signaling axis, thereby preventing YAP/TAZ phosphorylation. This facilitates the nuclear translocation of YAP/TAZ, where they bind to TEAD transcription factors, ultimately upregulating osteogenic genes such as *RUNX-2*, *COL-1* and *OCN*, thereby driving the osteogenic differentiation process.

### Proliferation and *in vitro* osteoclast differentiation of RAW264.7 cells on four surface-modified materials

**Figure 5:**
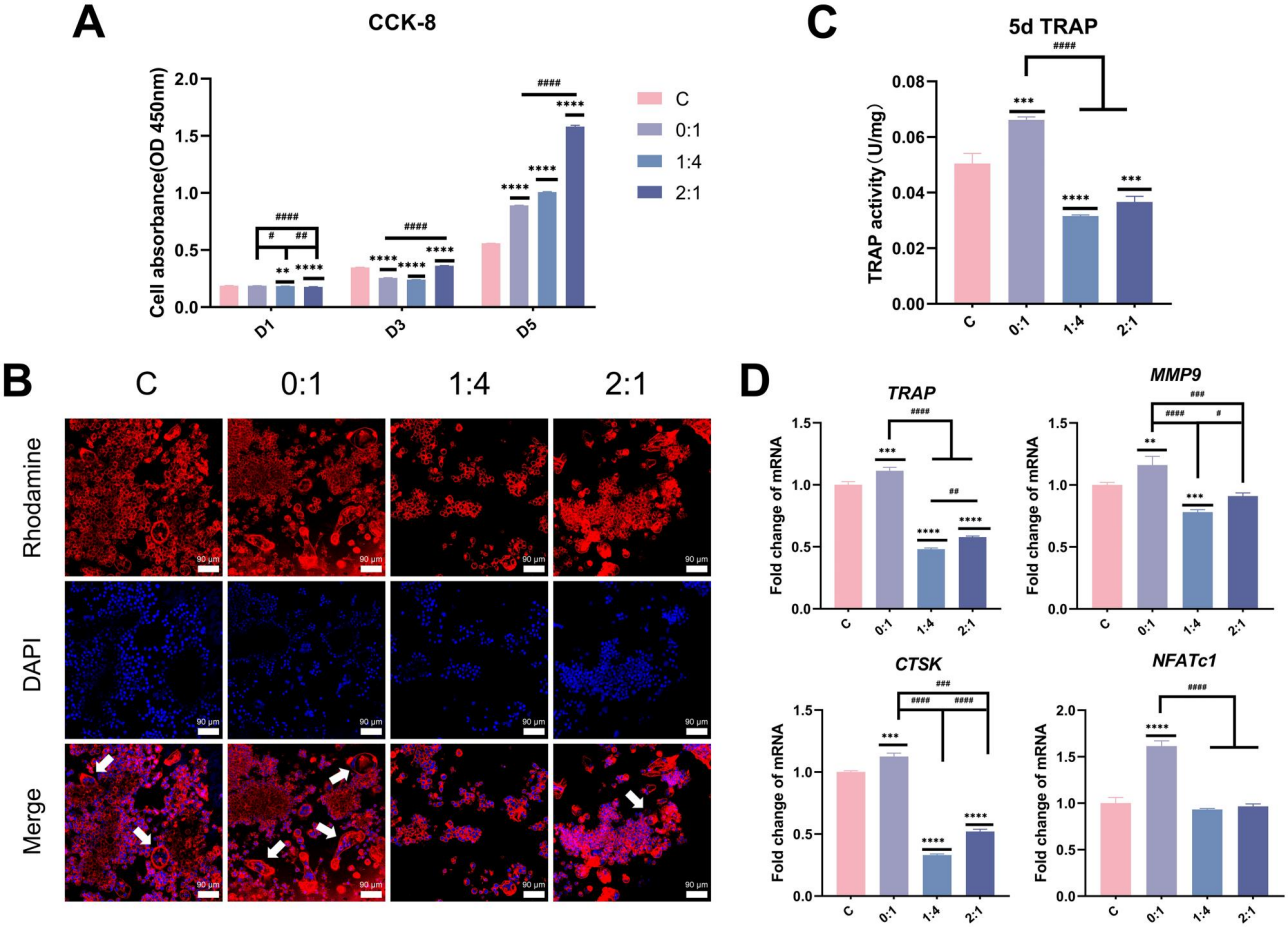
Effects of C, 0:1, 1:4 and 2:1 materials on RAW264.7 cell proliferation and *in vitro* osteoclast differentiation. (**A**) Cytotoxicity assay via CCK-8 assay. (**B**) Cell morphology observed by confocal laser scanning microscopy after 5 days of induction. (**C**) Quantitative TRAP activity assay and (**D**) relative expression levels of osteoclast differentiation-related genes after 5 days of induction. Data are presented as mean ± SD (*n* = 3). *P < 0.05, **P < 0.01, ***P < 0.001, ****P < 0.0001 vs. the C (CFR-PEEK) control group; #*P *< 0.05, ##*P *< 0.01, ###*P *< 0.001, ####*P *< 0.0001 among the 0:1, 1:4 and 2:1 groups.

As shown in Figure 5A, the CCK-8 assay revealed that acid-treated materials significantly promoted RAW264.7 cell proliferation from Days 1 to 5. Cell growth exhibited marginal variations across groups during the first three days. By Day 5, cell proliferation in all experimental groups exceeded that of the control group (*P *< 0.05), following the pattern: 2:1 > 1:4 > 0:1 > C. This indicates that the acid treatment was nontoxic to cells and possessed a favorable proliferative effect. Confocal microscopy (Figure 5B) revealed all groups contained multinucleated cells (≥3 nuclei) after 5 days, confirming osteoclast presence. The 0:1 group exhibited the highest density and largest size of actin rings, followed by the C group. The 2:1 group showed sparser rings, and notably, no distinct actin rings were observed in the 1:4 group, suggesting a pronounced inhibition of osteoclast differentiation. As demonstrated in Figure 5C, TRAP activity was significantly higher in the 0:1 group than in the control (*P *< 0.05); conversely, it was significantly suppressed in the 1:4 (*P *< 0.0001) and 2:1 (*P *< 0.001) groups. The 1:4 group exhibited the lowest activity, indicating the most potent inhibition of osteoclastogenesis. RT-qPCR analysis (Figure 5D) revealed that the expression levels of osteoclast-related genes (*TRAP, MMP9, CTSK* and *NFATC1*) followed the order: 1:4 < 2:1 < C < 0:1. The 0:1 group showed a significant upregulation (*P *< 0.05) indicative of pro-osteoclast activity, while the 1:4 and 2:1 groups suppressed gene expression, with the 1:4 group exhibiting the strongest inhibition, consistent with confocal and TRAP results.

Therefore, the 1:4 ratio exhibited a significantly greater inhibitory effect on osteoclast differentiation at both the genetic and functional levels, followed by the 2:1 ratio.

### *In vitro* angiogenesis of HUVECs on four material surfaces

**Figure 6:**
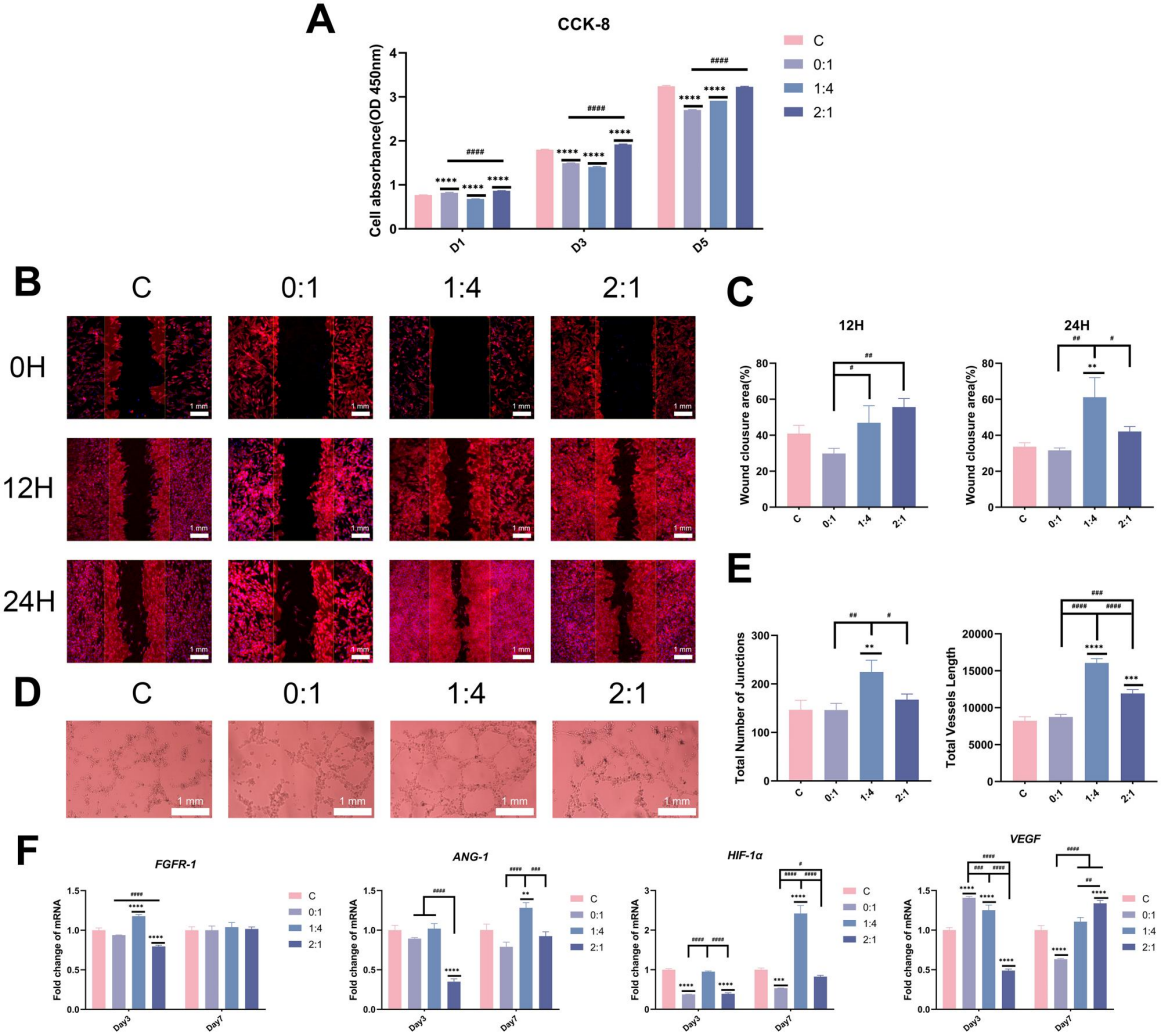
*In vitro* angiogenesis performance of HUVECs on C, 0:1, 1:4 and 2:1 material surface. (**A**) Cytotoxicity evaluated via CCK-8 assay. (**B**) Representative images and (**C**) quantitative analysis of HUVEC migration at 0, 12 and 24 h. (**D**) Representative tube formation images and (**E**) quantitative analysis of total vessel length and nodule count after 3 days of culture. (**F**) Relative expression levels of angiogenesis-related genes after 3 and 7 days of culture. Data are presented as mean ± SD (*n* = 3). *P < 0.05, **P < 0.01, ***P < 0.001, ****P < 0.0001 vs. the C (CFR-PEEK) control group; #*P *< 0.05, ##*P *< 0.01, ###*P *< 0.001, ####*P *< 0.0001 among the 0:1, 1:4 and 2:1 groups.

CCK-8 assays revealed (Figure 6A) that HUVEC viability in the 0:1, 1:4 and 2:1 group exceeded 70% of that of the control group at Days 1, 3 and 5, meeting the ISO 10993-5 biocompatibility requirements and indicating no significant cytotoxicity. The scratch assay (Figure 6B and C) demonstrated significantly enhanced HUVEC migration in the 1:4 and 2:1 groups. Notably, the 1:4 group achieved the most substantial wound closure at 24 h, with a healing rate of 61.17 ± 0.09%, significantly outperforming the other groups (*P *< 0.05). *In vitro* tube formation assays (Figure 6D and E) further demonstrated that the 1:4 group exhibited optimal performance in both lumen number and total length (*P *< 0.05). The overall angiogenic efficacy exhibited the following trend: 1:4 > 2:1 > 0:1 ≈ C. RT-qPCR results (Figure 6F) revealed that the 1:4 group showed the highest expression of vascular-related genes, including *FGFR-1*, *ANG-1* and *HIF-1α*, with significant temporal variations. The 0:1 and 2:1 groups exhibited comparatively high expression levels for specific genes (e.g., *VEGF*).

Collectively, the 1:4 group demonstrated optimal performance in promoting endothelial cell proliferation, migration and angiogenesis, exhibiting excellent angiogenic potential.

### *In vitro* antimicrobial activity of four material groups

**Figure 7:**
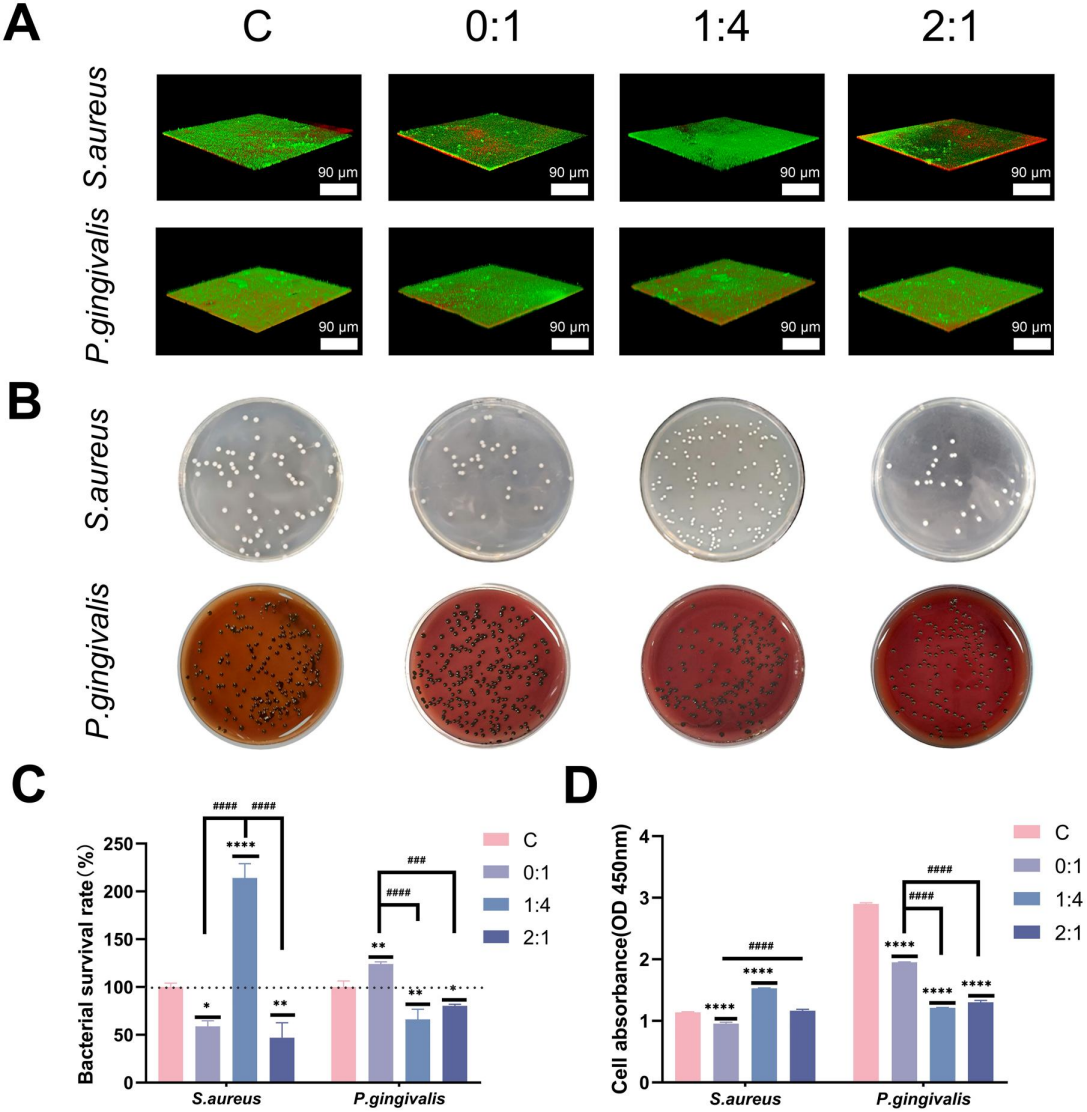
*In vitro* antibacterial activity of C, 0:1, 1:4 and 2:1 material against *S. aureus* and *P. gingivalis*. (**A**) Live/dead fluorescent staining of bacterial colonies on different material surfaces. (**B**) Representative photographs of colony-forming units (CFUs) on agar plates. (**C**) Quantitative analysis of bacterial survival rates based on CFUs. (**D**) Bacterial viability assessed via CCK-8 assay. Data are presented as mean ± SD (*n* = 3). *P < 0.05, **P < 0.01, ***P < 0.001, ****P < 0.0001 vs. the C (CFR-PEEK) control group; #*P *< 0.05, ##*P *< 0.01, ###*P *< 0.001, ####*P *< 0.0001 among the 0:1, 1:4 and 2:1 groups.

This study systematically evaluated the antimicrobial efficacy of various materials against *S. aureus* and *P. gingivalis* using colony-forming unit (CFU) counting, live/dead staining and biofilm quantification. As shown in Figure 7A–C, the CFU and live/dead staining results revealed distinct antibacterial profiles. The 2:1 group exhibited the strongest antibacterial effect against *S. aureus*, with colony counts reduced to approximately 50% of the control and a significantly increased proportion of dead (red-stained) bacteria. The 0:1 group exhibited a narrow spectrum effect, moderately inhibiting *S. aureus*, while the 1:4 group demonstrated negligible antibacterial activity against *S. aureus*. Furthermore, the 1:4 and 2:1 groups exhibited optimal antibacterial effects against *P. gingivalis*, showing significantly lower CFU counts than the control (*P *< 0.05) and reduced viable bacterial ratios. Notably, the 0:1 group yielded no significant antibacterial effect against *P. gingivalis*. The CCK-8 assay results (Figure 7D) further revealed that the 0:1 group demonstrated optimal efficacy against *S. aureus* biofilm (*P *< 0.05), while the 2:1 group exhibited no significant difference from the control. For *P. gingivalis* biofilm, all acid-treated groups exhibited significant inhibition compared to the control (*P *< 0.05). In this respect, the 1:4 and 2:1 groups demonstrated the most potent inhibitory effects, which were significantly superior to those observed in the 0:1 group.

In summary, the 2:1 group demonstrated superior overall performance characterized by potent broad-spectrum antibacterial activity and effective biofilm control. The 1:4 group exhibited significant antibacterial effects against *P. gingivalis*, while the 0:1 group showed specific inhibitory effects against *S. aureus* and its biofilm.

### *In vivo* osteogenic potential assessment

**Figure 8:**
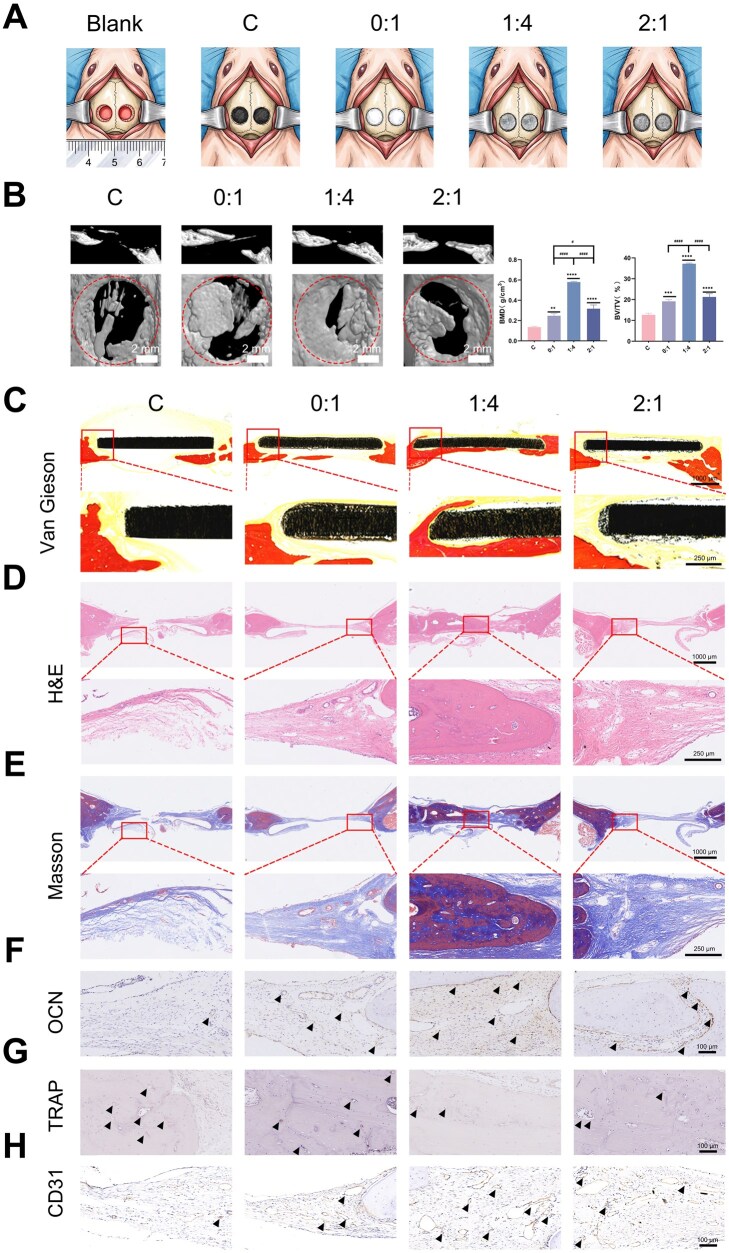
*In vivo* osseointegration evaluation of the C, 0:1, 1:4 and 2:1 materials in a rat cranial defect model. (**A**) Schematic diagram of material implantation for each group. (**B**) Representative micro-CT reconstructions and quantitative analyses of bone mineral density (BMD) and bone volume/tissue volume (BV/TV) at 8 weeks post-implantation. (**C**) Van Gieson, (**D**) H&E and (**E**) Masson’s trichrome staining of the defect sites. (**F**) Immunohistochemical staining for Osteocalcin (OCN); arrows indicate distinct positive granules in the cytoplasm/perinuclear region. (**G**) TRAP staining; arrows indicate TRAP-positive multinucleated osteoclasts. (**H**) Immunohistochemical staining for CD31 marking neovascularization; arrows indicate positive expression. Data are presented as mean ± SD (*n* = 3 animals, 6 implants). *P < 0.05, **P < 0.01, ***P < 0.001, ****P < 0.0001 vs. the C (CFR-PEEK) control group; #*P *< 0.05, ##*P *< 0.01, ###*P *< 0.001, ####*P *< 0.0001 among the 0:1, 1:4 and 2:1 groups.

A rat cranial defect model was established to evaluate bone regeneration, with a schematic of the surgical implantation sites presented in Figure 8A. All animals survived the 8-week postsurgical period, with no significant inflammation observed at the implantation sites, and the materials remained stably positioned within the bone defect areas. Micro-CT analysis (Figure 8B) at 8 weeks post-implantation revealed significant differences in bone regeneration. The 1:4 group exhibited the highest bone mass, with a BMD of 0.58 ± 0.01 g/cm^3^ and BV/TV of 37.21 ± 0.36%, significantly exceeding the control values (0.14 ± 0.01 g/cm^3^ and 12.64 ± 0.84%, respectively). Van Gieson (VG) staining results (Figure 8C) further demonstrated that the material-bone interface in Group C was smooth, showing only sparse, loose fibrous connective tissue with minimal new bone formation. The 0:1 group presented more new bone formation than Group C. Meanwhile, the 2:1 group displayed denser fibrous tissue at the material interface. Notably, the 1:4 group showed the most compact newly formed bone, which tightly adhered to the material surface and achieved the best osseointegration. HE staining results (Figure 8D) showed that the areas of eosinophilic osteoid proliferation were larger in all experimental groups (0:1, 1:4 and 2:1) compared to the control group, with the 1:4 group exhibiting the largest area. Masson’s trichrome staining (Figure 8E) further demonstrated that collagen fiber deposition (blue) was significantly more pronounced in all experimental groups compared to the control group, with the 1:4 group exhibiting the densest and ordered collagen arrangement, alongside the highest proportion of mature bone tissue (red). As shown in Figure 8F–H, the 1:4 group exhibited optimal performance across osteogenic, osteoclastic and angiogenic markers. This group demonstrated the strongest OCN-positive signal, indicating the highest late-stage osteogenic activity. Moreover, it contained the fewest TRAP-positive osteoclasts (characterized by burgundy cytoplasm and light blue nuclei), indicating effective inhibition of bone resorption. CD31 expression (dark brown granules) was also significantly higher than in other groups, suggesting the most active angiogenesis.

*In vivo* results demonstrated that the 1:4 group exhibited the best performance in promoting bone regeneration, enhancing osseointegration, facilitating collagen deposition and angiogenesis and suppressing osteoclast activity. The 2:1 and 0:1 groups also significantly outperformed the control group in all metrics, further validating their superior osteogenic properties.

## Discussion

CFR-PEEK was selected as the matrix material for this investigation, building upon its recognized suitability for orthopedic and dental applications derived from favorable mechanical properties [40]. By adjusting the volume ratio of nitric acid to concentrated sulfuric acid, four types of micro-to-nano surface topographies were constructed: three-dimensional porous (0:1), mildly etched (1:0, 4:1), nanoporous (1:1, 1:2 and 2:1) and nanocluster-composite porous (1:4). For specimens treated with the 1:4 acid ratio, the introduction of a minor quantity of nitric acid induced the concentrated sulfuric acid to preferentially form a micron-scale porous skeleton. Subsequently, a synergistic interaction with the nitric acid fragmented the surface into uniformly stacked nanoclusters, introducing hydrophilic sulfonic acid groups and nitro groups. The resulting hierarchical composite structure is advantageous for bone regeneration, vascular regulation and selective antibacterial activity [41]. The 2:1 group exhibited the most uniform pore size and the highest density of nanopores, indicating that a moderate nitric acid concentration optimally refines porosity without excessive corrosion. Monosulfuric acid (0:1) treatment produced a uniform three-dimensional porous network, significantly enhancing roughness and morphological uniformity. Conversely, mononitric acid (1:0) caused only minor corrosion without altering surface topology [42]. XPS and FTIR analysis confirmed the successful introduction of sulfonic and nitro groups via mixed-acid treatment. After hydrothermal washing [43], the sulfur content decreased to 0.22–0.38%, accompanied by weakened sulfonic acid absorption peaks. Concurrently, the nitrogen content increased to 3.59–5.78%, suggesting a possible partial substitution of sulfonic acid groups by nitro groups, which is consistent with previous literature [31]. Mechanical testing indicated that the elastic modulus of all samples remained within the human bone range (0.1–20 GPa) [44], while the compressive strength decreased by <7%, readily meeting ISO 5833 requirements (≥70 MPa). This indicated that the acid etching process was confined to the surface layer and did not disrupt the carbon fiber-matrix interface. All modified groups exhibited a hydrophobic-to-hydrophilic transition (contact angle decreasing from >90° to <90°), with the 1:4 ratio showing optimal hydrophilicity, followed by the 2:1 ratio. Related studies have demonstrated that the introduced polar groups [45, 46], including -SO_3_H and -NO_2_, can significantly reduce the water contact angle on material surfaces and render the zeta potential highly electronegative. These polar groups strengthen interfacial bonding strength via hydrogen bonding and polar interactions, thereby improving the physicochemical stability of the surface [47]. The combination of enhanced polar functional groups and the capillary forces generated by high roughness [48] provided additional anchorage sites for cell adhesion, osteogenesis and angiogenesis, laying a solid foundation for subsequent biological functional validation [49]. Notably, CFR-PEEK is inherently bio-inert; while it does not undergo bulk degradation [50, 51], its acid-etched surface may release trace ions via initial ion exchange [52]. A 28-day pH monitoring showed that all groups maintained stable pH values within the normal range, with only minor fluctuations. These trivial changes resulted from transient surface ion exchange rather than degradation, confirming excellent surface stability and biosafety for oral implants [53, 54].

### Effects of micro-to-nano topographies on osteogenic differentiation and underlying mechanisms

CCK-8 assays and confocal microscopy demonstrated that all acid-etched micro-to-nano surface topographies were thoroughly deacidified and significantly promoted the proliferation of MC3T3-E1 cells without cytotoxicity. The 0:1 group exhibited optimal cell spreading, while the 1:4 and 2:1 groups showed the highest adhesion cell densities with marked pseudopod extension. Surface roughness, specific surface area and hydrophilic functional groups collectively enhance the adsorption of serum proteins, including osteogenic proteins such as fibronectin and vitronectin, thereby providing a critical medium for cell adhesion [55–57]. Concurrently, the microstructure/nanostructure provides physical confinement, increases effective surface area and promotes integrin clustering. These chemical and physical cues act synergistically to accelerate focal adhesion maturation, thereby boosting the early osteogenic response [58]. Specifically, the three-dimensional interconnected channels of the 0:1 and 2:1 groups mimicked the native bone microenvironment, promoting three-dimensional cell spreading and migration [59]. Moreover, the surface nanoclusters in the 1:4 group (<100 nm) corresponded well to the scale of cellular pseudopods, thereby enhancing the ‘anchoring’ effect. This interaction activated the integrin-FAK-MAPK signaling pathway, synergistically boosting cell adhesion and proliferation capacity [60, 61]. This finding aligned with the study by Mei *et al.* [62], who reported that hierarchical surfaces increased bone marrow mesenchymal stem cell adhesion rates by approximately 30% compared to monolithic (single-layer) architecture.

ALP and ARS staining, along with osteogenic gene expression (*RUNX-2*, *COL-1*, *ALP*, *OCN* and *OPN*), demonstrated superior osteogenic potential in the 0:1, 1:4 and 2:1 groups compared to the control. The 2:1 group exhibited the highest ALP activity, indicating active early-stage osteogenesis, while the 1:4 group showed the most pronounced calcific nodules, reflecting the strongest terminal mineralization capacity [63]. Structure–property correlation analysis revealed that the three-dimensional porous structure (0:1) facilitated cellular support and migration, while the uniform nanopore layer (2:1) enhanced protein adsorption and signal transduction [30, 31]. In contrast, the micropore-nanocluster composite (1:4) produced a synergistic scale effect that significantly enhanced bone matrix mineralization by activating key pathways, such as Wnt/β-catenin, AKT and AMOT130/YAP, while also regulating the bone immune microenvironment [64, 65]. RNA-seq and subsequent Western blot/RT-qPCR analyses further revealed that the 1:4 micro- and nano-topology promoted osteogenesis via the Hippo-YAP/TAZ pathway. Upon sensing the topological cues, cells activate RhoA-mediated actin cytoskeleton remodeling, while subsequently suppress the SAV1/LATS1 complex, inducing YAP/TAZ dephosphorylation and nuclear translocation [66]. Within the nucleus, YAP and TAZ form complexes with TEAD transcription factors, upregulating target genes including *CYR61*, *CTGF*, *FN1* and *Serpine1* [67, 68], thereby continuously promoting *RUNX-2*, *COL-1* and *OCN* expression [69, 70]. Interestingly, the 1:4 group induced moderate inhibition of the LATS1/SAV1 complex, significantly enhancing YAP/TAZ accumulation and transcriptional activity. In contrast, the 0:1 and 2:1 groups may have exhibited a state of ‘excessive inhibition’, characterized by only limited upregulation of downstream target genes [71]. Immunofluorescence imaging confirmed that the 1:4 group significantly induced YAP nuclear translocation in MC3T3-E1 cells, acting as a key mechanotransduction mediator closely associated with osteoblast differentiation.

Furthermore, studies have indicated that YAP/TAZ nuclear translocation upregulates *CXCL12* and *VEGF-A*, promoting vascular-osteogenic coupling [72, 73]. Simultaneously, it modulates the *RANKL*/*OPG* ratio to suppress osteoclast activity, maintaining osteogenic-osteoclastic balance [70]. In summary, the micro- and nano-hierarchical structure of the 1:4 group achieved its significant pro-osteogenic effects by precisely modulating the Hippo-YAP/TAZ pathway, creating a molecular environment conducive to cell adhesion, proliferation and differentiation.

### Effects of micro-to-nano materials on osteoclast differentiation

It is well-established that bone regeneration relies on a dynamic equilibrium between osteogenic deposition and osteoclastic resorption. Building upon the previously established osteogenic ratios (0:1, 1:4 and 2:1), this study further evaluated their impact on osteoclastogenesis. The microcomposite and nanocomposite structure of the 1:4 group exerted the most potent inhibition, with cells maintaining a round, macrophage-like morphology without forming typical actin rings even after 5 days. In this respect, TRAP protein levels and expression of multiple osteoclast-related genes (*TRAP*, *MMP9*, *CTSK* and *NFATc1*) were significantly lower than in other groups, demonstrating the most potent inhibitory effect. Although the 2:1 group exhibited a small number of actin rings, osteoclast gene expression remained low, showing the second-strongest inhibitory effect. Conversely, the 0:1 group exhibited a pro-osteoclastic phenotype, characterized by the upregulation of *NFATc1* and its downstream genes, which promote osteoclast differentiation [74]. Nanoscale surfaces (1:4 and 2:1 groups) simultaneously upregulated the osteogenic genes (*RUNX-2* and *OPN*) while suppressing osteoclast-related genes, establishing a microenvironment conducive to heterotopic bone formation [75]. This dual regulation primarily achieves the inhibition of osteoclast differentiation via the MAPK (ERK/JNK) [76, 77] and NFATc1 signaling pathways [78]. Morphological analysis revealed that the nanostructured surfaces could promote the orderly alignment of small, regular multinucleated cells along large pore walls, whereas submicron surfaces tended to generate large, irregularly shaped osteoclasts. Chen *et al.* [79] also confirmed that while both microscale and nanoscale HA scaffolds exhibit osteoinductivity, nano-featured surfaces inhibit actin ring formation, thereby weakening osteoclast differentiation. Conversely, (sub)micron surfaces exhibit marked cytoplasmic expansion and tight matrix adhesion, forming the ‘ruffled border’ cytoskeleton essential for mature osteoclasts. Accordingly, while pure micrometer-scale structures (0:1 group) support osteogenesis, they concurrently stimulate osteoclast activity.

As discussed in section ‘effects of micro-to-nano topographies on osteogenic differentiation and underlying mechanisms’, the strong inhibitory effect of the 1:4 micro- and nano-hierarchical structure on osteoclastogenesis also relies on the dual regulation of TAZ, a key effector of the Hippo pathway. TAZ not only directly blocks osteoclast differentiation in osteoclast precursors by inhibiting the TAK1/NF‑κB pathway but also upregulates OPG and downregulates RANKL expression in osteoblasts to alter the local RANKL/OPG ratio [80]. This weakens the ability of mononuclear/macrophage lineage cells to receive critical differentiation signals, thereby synergistically suppressing bone resorption.

In addition to the aforementioned molecular regulation, material surface roughness serves as a key physical factor affecting osteoclast behavior. Due to the significant size differences between osteoblasts (≈10 µm diameter) and osteoclasts (20–100 µm diameter), micro-rough surfaces can promote osteoblast spreading while inhibiting osteoclast focal adhesion formation. Costa *et al.* [81] reported that nano-HA with *Ra *= 363 nm enhanced osteogenic activity and inhibited osteoclast adhesion, whereas smooth HA with *Ra *= 194 nm increased osteoclast adhesion rates by 2.5-fold. Davison *et al.* [82] reported that TRAP activity was twice as high on the surface with *Ra *= 0.126 µm compared to one with *Ra *= 1.287 µm. In this study, the 0:1 group exhibited a relatively smooth roughness (*Ra *= 143.00 ± 7.81 nm), which favored osteoclast formation. In contrast, the 1:4 and 2:1 groups exhibited roughness values of 522.67 ± 53.15 nm and 364.00 ± 40.58 nm, respectively, significantly inhibiting osteoclast adhesion.

In summary, the 1:4 micro- and nano-hierarchical structure exerts synergistic effects at both molecular signaling (TAZ, NFATc1 and MAPK) and surface roughness levels, significantly inhibiting osteoclast differentiation and bone resorption while enhancing osteogenic activity, thereby providing an ideal microenvironment for functional bone regeneration.

### Effects of micro-to-nano materials on vascularization

Angiogenesis is a critical prerequisite for early bone tissue regeneration and interface integration following implant placement. An adequate vascular network facilitates nutrient supply, oxygen exchange and the removal of metabolic waste within the local microenvironment, thereby accelerating osteoblast proliferation and differentiation while promoting rapid integration at the bone-implant interface [83]. This study systematically evaluated the regulatory effects of different micro-to-nano surface topographies on angiogenesis across three dimensions: tube formation capacity, cell migration behavior and expression of vascular-related genes. *In vitro* migration and tube formation assays revealed that endothelial cells in the 1:4 group exhibited the strongest directional movement into the ischemia-simulated zone. This group formed the highest number of tubular structures with the greatest total length, featuring a continuous and uniform luminal network. The cells adopted elongated, polarized morphologies, indicating advanced stages of angiogenesis. At the gene expression level, the 1:4 group showed significant upregulation of key angiogenic genes, including *FGFR-1*, *ANG-1* and *HIF-1α*. Subsequent *in vivo* experiments further demonstrated higher CD31 protein expression in this group, confirming its robust pro-angiogenic capacity at both the transcriptional and translational levels. In contrast, while the 0:1 and 2:1 groups exhibited mild pro-angiogenic capacity, they generated fewer lumens, shorter migration distances and a more limited gene expression response compared to the 1:4 group.

As discussed in section ‘effects of micro-to-nano topographies on osteogenic differentiation and underlying mechanisms’, YAP and TAZ—the key effectors of the Hippo pathway—act as core transcriptional co-activators and regulate the transcription of multiple endothelial function-related genes by binding to TEAD transcription factors. Klaihmon *et al.* [84] confirmed that knockdown of YAP or TAZ in endothelial progenitor cells significantly downregulates VEGFA transcription, impairs endothelial cell function and inhibits angiogenesis. This suggests that the 1:4 microstructure-nanostructure can provide intrinsic molecular support for vascularization by regulating the YAP/TAZ signaling axis.

Building upon this molecular mechanism, the pro-angiogenic effects of microcomposite and nanocomposite structures also stem from the synergistic interactions of multiple physical parameters. Physical characteristics such as pore size distribution, connectivity and porosity on material surfaces collectively regulate cell migration, proliferation and vascular network formation [85]. It is increasingly recognized that multiscale surface topographies influence the angiogenic microenvironment through distinct biophysical and immunomodulatory mechanisms [86]. Compared to single-scale structures, micro- and nano-hierarchical composites promote angiogenesis by directly stimulating endothelial cells and indirectly regulating macrophage phenotype. Furthermore, the incorporation of nanoscale features enhances cell-cell communication and paracrine signaling, perfectly complementing the mechanical support provided by micrometer-scale scaffolds [87, 88]. Concurrently, the hierarchical porous networks facilitate more stable nutrient-oxygen diffusion gradients, thereby enhancing mass transport and maintaining metabolic homeostasis. This is highly consistent with prior research: Bai *et al.* [89] reported that microcomposite and nanocomposite surfaces enhanced angiogenesis more effectively than single-scale nanostructures, and Yang *et al.* [87] developed novel bioceramic topographies exhibiting superior coupled osteogenic and angiogenic properties.

In summary, the 1:4 micro- and nano-hierarchical structure effectively promotes vascular network formation and functional maturation due to its unique physical and topological characteristics, creating favorable conditions for rapid vascularization at the material-tissue interface. Combined with its outstanding performance in regulating osteogenesis and osteoclastogenesis, this structure demonstrates significant application value in the design of functional bone regeneration materials.

### Effects of micro-to-nano materials on bacterial inhibition

Implant-associated infections significantly impact the osseointegration process, with *S. aureus* and *P. gingivalis* being the most common pathogenic microorganisms. *S. aureus* is a Gram-positive bacterium that possesses a 20–80 nm thick peptidoglycan layer without an outer membrane, which confers high mechanical strength and a propensity for biofilm formation, making it a primary pathogen in early infections [90]. Conversely, *P. gingivalis* is a Gram-negative bacterium with a thin peptidoglycan layer (only 10–15 nm thick) situated between an inner membrane and an outer membrane rich in lipopolysaccharides (LPS). It exhibits weak mechanical stability and is frequently implicated in periodontitis and secondary bone resorption [91, 92]. These inherent structural differences provide a theoretical basis for utilizing surface engineering to achieve bacteria-specific regulation.

This study revealed a distinct, bacteria-specific antibacterial profile for the different surface topographies. Against *S. aureus*, the 0:1 (micropore) and 2:1 (nanopore) groups both significantly inhibited bacterial adhesion, whereas the 1:4 (nanocluster-composite) group showed no obvious inhibition but even slightly promoted adhesion. In contrast, for *P. gingivalis*, the 1:4 group displayed the strongest inhibitory effect, followed by the 2:1 group, while the 0:1 group significantly promoted bacterial adhesion.

This selective antibacterial behavior can be elucidated by surface charge characteristics introduced during acid etching. As shown in Figure 2E, the 1:4 etching system generated a higher density of polar groups (e.g., nitro or sulfonic groups) than the 0:1 and 2:1 groups, resulting in a more negatively charged surface. Gram-negative *P. gingivalis* possesses a strongly negatively charged LPS outer membrane, which experiences intense electrostatic repulsion from the 1:4 surface, severely inhibiting its initial adhesion and compromising its outer membrane stability [93]. In contrast, Gram-positive *S. aureus* lacks an outer membrane and has a thick peptidoglycan layer with low surface charge density, making it less sensitive to electrostatic repulsion [94]. This distinct structural vulnerability explains the lack of prominent inhibition against *S. aureus* by the 1:4 group.

Mechanistically, in the 0:1 and 2:1 groups, the surface depression size was comparable to the diameter of *S. aureus*, which limited the effective contact area and physically hindered stable adhesion. In the 1:4 group, the surface nanoclusters provided additional anchor points, inadvertently enhancing the capture of *S. aureus*, which is consistent with Meinshausen *et al.* [95] report that specific configurations of microscale and nanoscale structures can enhance bacterial capture. However, for *P. gingivalis*, the nanoclusters in the 1:4 group synergistically inhibited bacterial adhesion through two complementary mechanisms: physical disruption (nano-piercing) of its thin outer membrane [96] and electrostatic repulsion between the negatively charged surface and the LPS. The 2:1 group exhibited a secondary antibacterial effect against *P. gingivalis*, primarily by reducing the contact area. In contrast, the microporous protrusions of the 0:1 group formed a micrometer-scale geometric match with bacterial fimbriae and capsules [97, 98], providing specific adhesion sites that actively promoted colonization.

Surface roughness and hydrophilicity were also identified as critical physical factors influencing bacterial behavior. The high surface roughness (*Ra* > 0.5 µm) of the 1:4 group increased the actual contact area with *S. aureus*, while locally elevated surface energy reduces adhesion activation energy, thereby facilitating its adhesion [99]. Conversely, *P. gingivalis* exhibited greater sensitivity to the water contact angle. Its adhesion is typically inhibited by the ‘air bubble effect’ at contact angles >150° and by thick water film formation at <30° [100, 101]. The 0:1 group (contact angle ≈75°, weakly hydrophilic) provided the most permissive conditions for *P. gingivalis* adhesion.

In summary, the single-scale microporous or nanoporous structures (0:1 and 2:1 groups) are particularly suitable for inhibiting thick-walled, spherical *S. aureus* by restricting its contact area. Meanwhile, the 1:4 microcomposite and nanocomposite structure achieves potent and selective inhibition against the Gram-negative, rod-shaped *P. gingivalis* through the synergistic effects of surface charge repulsion, nanostructure-mediated mechanical puncturing and membrane disruption.

### Effects of micro-to-nano materials on *in vivo* bone integration

Bone tissue primarily forms through two pathways: intramembranous ossification and endochondral ossification. Intramembranous ossification primarily contributes to the development of flat bones (e.g., craniofacial bones and clavicle), where mesenchymal stem cells directly or indirectly differentiate into osteoblasts and deposit bone matrix. In contrast, endochondral ossification governs the formation of long bones (e.g., the tibia and femur), involving a gradual replacement process from mesenchymal cells to cartilage templates and ultimately to bone tissue [102, 103]. The rat cranial defect model was selected for this experiment because both the cranium and the mandible originate via intraperiosteal ossification during embryogenesis and share a similar ‘cortical bone-cancellous bone-cortical bone’ dual-layer architecture with analogous repair mechanisms. This model offers a robust platform for evaluating the biological efficacy of CFR-PEEK composites in dental and maxillofacial bone defect repair.

Micro-CT, VG, H&E and Masson’s trichrome staining, alongside immunohistochemical results for OCN, TRAP and CD31, indicated that the 1:4 group (nanoclusters-composite porous) significantly outperformed the control, 0:1 (microporous) and 2:1 (nanoporous) groups across all evaluation metrics. Specifically, BMD and BV/TV were significantly enhanced, resulting in near-complete defect closure. The collagen fiber arrangement exhibited superior density and organization. The late-stage osteogenic marker OCN showed high-density positivity in the 1:4 group, indicating the highest level of terminal mineralization. Moreover, TRAP staining revealed the fewest osteoclasts in this group, confirming its ability to inhibit osteoclast activation *in vivo* by disrupting actin ring formation and regulating the RANKL/OPG ratio [104]. CD31 immunohistochemistry further confirmed the most robust angiogenic response in the 1:4 group [105], which is highly consistent with reports by Xing *et al.* [106] demonstrating that functionalized nanoclusters and porous composite structures enhance endothelial cell activity, VEGF secretion and CD31 expression.

An analysis of structure–function relationships revealed that the 0:1 and 2:1 groups provided high specific surface areas to promote early cell adhesion, consistent with Davison *et al.* [82] findings that microporous scaffolds enhance osteogenic differentiation, with their single pore size limited vascular channel formation and impeding late-stage bone matrix maturation. In the present study, the 1:4 group exhibited a hierarchical pore structure spanning the nanoscale to macroscale. This architecture formed a 3D network that closely mimicked the natural extracellular matrix, providing ample adhesion sites while creating effective channels for vascular permeation. Furthermore, the sulfonic and nitro groups generated by the mixed-acid etching may slowly release trace amounts of H^+^, SO_4_^2-^ and NO_3_^-^, delicately regulating the local ion homeostasis without inducing acidosis, thereby promoting collagen cross-linking and mineralization [13]. This structural effect mirrors the enhanced bone density achieved by Zhang *et al.* using HA-BC nanocluster-composite scaffolds, further validating the distinct advantages of hierarchical topographies in tissue regeneration [107].

In summary, the nanoclusters-composite three-dimensional porous structure (the 1:4 group) achieves comprehensive advantages through mechanochemical synergy, including maximized osteoblast differentiation, minimized osteoclast activation and the most pronounced vascularization. While the 0:1 sulfonated micropores enhance protein adsorption, their long-term osteogenic and angiogenic effects remain limited. Conversely, 2:1 nanopores facilitate early collagen deposition but exhibit restricted vascular permeability due to their smaller pore size. This study demonstrated that CFR-PEEK hybrid acid-etched materials could significantly promote early osseointegration.

### Limitations and future perspectives

Despite these promising findings, this study has several limitations that warrant further investigation. First, while the rat cranial defect model shares the intramembranous ossification mechanism with jaw bones (making it suitable for preliminary intrinsic bioactivity evaluation), it differs biomechanically from the alveolar bone environment, where dental implants are subjected to complex masticatory loads. Future studies utilizing large animal mandibular models (e.g., beagle dogs) are necessary to evaluate osseointegration under functional loading and polymicrobial infection conditions [108]. Second, while the 1:4 group demonstrated excellent short-term stability, the potential release of wear debris from the modified porous layer under long-term *in vivo* loading (>6 months) remains a concern. Further investigation is required to further clarify whether such wear debris eventually shifts macrophage polarization from the pro-healing M2 phenotype toward the inflammatory and osteolytic M1 phenotype [109]. Finally, although we identified the Hippo-YAP/TAZ axis as a key regulator via multilevel analyses, direct functional validation through pathway inhibitor/activator rescue assays is still lacking. The potential crosstalk between this axis and other mechanotransduction cascades warrants comprehensive future exploration.

Nevertheless, the modified CFR-PEEK developed in this work exhibits profound application potential in oral implantology, particularly for endosseous implantation in edentulous patients and alveolar bone augmentation [110]. Drawing upon existing literature, its utility can be logically extended to multiple oral applications, including implant abutments and fixed crowns/bridges. With intrinsic advantages such as a bone-matching elastic modulus, MRI compatibility and favorable esthetics, it provides a highly promising alternative for oral restoration [111]. Moving forward, addressing the aforementioned limitations—through long-term functional loading assessments, mixed-species biofilm evaluations and molecular rescue verifications—will be critical to validating its adaptability and safety in real-world clinical environments, ultimately facilitating its translational success.

## Conclusions

To overcome the inherent bio-inertness of CFR-PEEK, this study employed a facile, one-step nitric-sulfuric acid (1:4) etching strategy to construct a unique ‘nanocluster/3D porous network’ hierarchical structure on its surface. This optimized topography comprehensively enhances early osseointegration by precisely regulating the Hippo-YAP/TAZ signaling pathway to stimulate osteogenesis and angiogenesis, while simultaneously suppressing osteoclast activity. Furthermore, this surface exhibits highly selective antibacterial efficacy against the Gram-negative periodontal pathogen *P. gingivalis* via the synergistic effects of electrostatic repulsion and nanostructure-mediated membrane disruption.

In summary, this straightforward and cost-effective surface modification technique successfully achieves the integrated optimization of osteogenesis, angiogenesis, anti-osteoclastogenesis and selective antibacterial performance. From a broader perspective, the micro- and nano-hierarchical CFR-PEEK exhibits immense potential for clinical implant restoration and craniofacial bone repair, offering a valuable referential paradigm for the rational design of next-generation multifunctional polyetheretherketone-based biomaterials.
